# LIVING ARRANGEMENTS, HOUSING CHARACTERISTICS, AND PSYCHOLOGICAL WELL-BEING AMONG OLDER ADULTS IN CHINA

**DOI:** 10.1093/geroni/igad104.2348

**Published:** 2023-12-21

**Authors:** Rong Fu

**Affiliations:** Siena College, Loudonville, New York, United States

## Abstract

**Objectives:**

Living arrangements is an essential component of social environments for older adults. This study examines the impact of living arrangements and housing characteristics on the psychological well-being of older adults in China.

**Methods:**

Data were derived from the 2011-2012 wave of the Chinese Longitudinal Healthy Longevity Survey. The final sample consisted of 4,746 Chinese older adults aged 80–105 years. Multivariate logistic regression models were used to estimate the odds of negative psychological outcomes, predicted by living arrangements, housing characteristics and other covariates.

**Results:**

Overall, older adults who lived in a three-person household were less likely to feel lonely than their counterparts living in other household sizes. Participants who reported at least one housing problem (e.g., exposure to mold, minor home damage) were more likely to feel anxious and depressed than those who did not report any housing problems. Having a private bedroom was associated with better psychological well-being for both older men and women.

**Discussion:**

This study demonstrates that both household sizes and housing conditions may influence health and well-being in advanced age. Living with family members and having adequate personal space at home have protective health benefits for older adults. This study has policy implications regarding household sizes and improvement in housing conditions.

